# Improved labelling of DTPA- and DOTA-conjugated peptides and antibodies with ^111^In in HEPES and MES buffer

**DOI:** 10.1186/2191-219X-2-4

**Published:** 2012-01-27

**Authors:** Maarten Brom, Lieke Joosten, Wim JG Oyen, Martin Gotthardt, Otto C Boerman

**Affiliations:** 1 Department of Nuclear Medicine, Radboud University Nijmegen Medical Centre, PO Box 9101, Nijmegen, 6500 HB, The Netherlands

**Keywords:** ^111^In-radiolabelling, peptides, antibodies, chelator

## Abstract

**Background:**

In single photon emission computed tomography [SPECT], high specific activity of ^111^In-labelled tracers will allow administration of low amounts of tracer to prevent receptor saturation and/or side effects. To increase the specific activity, we studied the effect of the buffer used during the labelling procedure: NaAc, NH_4_Ac, HEPES and MES buffer. The effect of the ageing of the ^111^InCl_3 _stock and cadmium contamination, the decay product of ^111^In, was also examined in these buffers.

**Methods:**

Escalating amounts of ^111^InCl_3 _were added to 1 μg of the diethylene triamine pentaacetic acid [DTPA]- and 1,4,7,10-tetraazacyclododecane-1,4,7,10-tetraacetic acid [DOTA]-conjugated compounds (exendin-3, octreotide and anti-carbonic anhydrase IX [CAIX] antibody). Five volumes of 2-(*N*-morpholino)ethanesulfonic acid [MES], 4-(2-hydroxyethyl)-1-piperazineethanesulfonic acid [HEPES], NH_4_Ac or NaAc (0.1 M, pH 5.5) were added. After 20 min at 20°C (DTPA-conjugated compounds), at 95°C (DOTA-exendin-3 and DOTA-octreotide) or at 45°C (DOTA-anti-CAIX antibody), the labelling efficiency was determined by instant thin layer chromatography. The effect of the ageing of the ^111^InCl_3 _stock on the labelling efficiency of DTPA-exendin-3 as well as the effect of increasing concentrations of Cd^2+ ^(the decay product of ^111^In) were also examined.

**Results:**

Specific activities obtained for DTPA-octreotide and DOTA-anti-CAIX antibody were five times higher in MES and HEPES buffer. Radiolabelling of DTPA-exendin-3, DOTA-exendin-3 and DTPA-anti-CAIX antibody in MES and HEPES buffer resulted in twofold higher specific activities than that in NaAc and NH_4_Ac. Labelling of DTPA-exendin-3 decreased with 66% and 73% for NaAc and NH_4_Ac, respectively, at day 11 after the production date of ^111^InCl_3_, while for MES and HEPES, the maximal decrease in the specific activity was 10% and 4% at day 11, respectively. The presence of 1 pM Cd^2+ ^in the labelling mixture of DTPA-exendin-3 in NaAc and NH_4_Ac markedly reduced the labelling efficiency, whereas Cd^2+ ^concentrations up to 0.1 nM did not affect the labelling efficiency in MES and HEPES buffer.

**Conclusions:**

We showed improved labelling of DTPA- and DOTA-conjugated compounds with ^111^In in HEPES and MES buffer. The enhanced labelling efficiency appears to be due to the reduced competitive chelation of cadmium. The enhanced labelling efficiency will allow more sensitive imaging of the biomarkers with SPECT.

## Introduction

Radiolabelled peptides and antibodies are used for molecular imaging and radionuclide therapy of tumours. The most successful example of peptide receptor imaging is the somatostatin analogue octreotide, which targets the somatostatin receptor subtype 2, overexpressed on neuroendocrine tumours. Tracers labelled with a radiometal via a chelator have the advantage that they can be labelled with high efficiency (> 95%) without the need for post-labelling purification and that the metabolites are trapped in the lysosomes of the cell, leading to higher accumulation in the target cell. This phenomenon is referred to as 'metabolic trapping' [1-5]. Ideally, low peptide or protein doses are administered because high doses may lead to saturation of the receptor, resulting in reduced accumulation of the radiotracer in the target tissue [6]. In addition, higher doses may cause toxic side effects, especially when agonists are used. In order to administer activity doses sufficient for imaging (single photon emission computed tomography or planar scintigraphy), tracers with a high specific activity [SA] are required. There is a need to further increase the SA to improve image quality, especially in the preclinical setting. In general, the tracer doses administered in rodent models must be kept low while at the same time administering relatively high activity doses (> 10 MBq/animal). ^111^In is a widely used radionuclide for the labelling of peptides and proteins used for imaging purposes. To enable labelling with a radiometal, such as ^111^In, the targeting molecule has to be conjugated with a chelator. The most commonly used chelators are diethylene triamine pentaacetic acid [DTPA] and 1,4,7,10-tetraazacyclododecane-1,4,7,10-tetraacetic acid [DOTA]. Labelling of DTPA- and DOTA-conjugated compounds is a one-step reaction in which the conjugated compound is incubated with ^111^InCl_3 _in a slightly acidic buffer, keeping the pH between 4 and 5.5. Acetate buffers are commonly used as a buffer for ^111^In-labelling of DTPA- and DOTA-conjugated compounds. Acetate buffers readily form coordination complexes with metals. It is assumed that coordinating buffers are needed for efficient chelation of radiometals [7]. However, for ^68^Ga-labelling of DOTA-conjugated compounds, 4-(2-hydroxyethyl)-1-piperazineethanesulfonic acid [HEPES] is successfully used as a buffer. Although developed for biological purposes by Good et al., HEPES has beneficial characteristics in chemistry involving metal ions as a non-coordinating buffer [8]. 2-(*N*-morpholino)ethanesulfonic acid [MES] was also described as a 'good buffer' [8] and has similar characteristics. Although HEPES and MES were described as non-coordinating buffers, recent reports showed that HEPES forms weak complexes with Cu(II) and Pb(II), but not with Zn(II) or Cd(II) [9,10]. Therefore, the term 'weakly coordinating' buffers seems to be more appropriate.

The fact that HEPES is successfully used for labelling of compounds with ^68^Ga prompted us to examine the effect of the weakly coordinating buffers, HEPES and MES, on the ^111^In-labelling and compared this with the radiolabelling in routinely used acetate buffer (sodium acetate and ammonium acetate). For comparison of the radiolabelling in these buffers, two peptides, exendin-3 and octreotide, and the chimeric monoclonal antibody [mAb] targeting carbonic anhydrase IX [CAIX], each conjugated with DTPA or DOTA, were used.

## Experimental procedures

### Peptides and antibodies and conjugation with DTPA or DOTA

DTPA-Tyr^3^-octreotide, DOTA-Tyr^3^-octreotide, [Lys^40^(DTPA)]exendin-3 [DTPA-exendin-3] and [Lys^40^(DOTA)]exendin-3 [DOTA-exendin-3] [11] were purchased from Peptide Specialty Laboratories GmbH (Heidelberg, Germany). The chimeric mAb anti-CAIX (cG250) was obtained from Wilex AG (Munich, Germany). The conjugation of anti-CAIX with SCN-Bz-DTPA or SCN-Bz-DOTA (Macrocyclics, Dallas, TX, USA) with a 50-fold molar excess was performed in a 0.1 M NaHCO_3 _buffer, with a pH of 8.2. After 1-h incubation, the conjugation mixture was dialyzed in a dialysis cell with a molecular cut-off value of 20 kD (Slide-a-lyzer, Pierce, Rockford, IL, USA) against 0.25 M ammonium acetate (pH 5.5) with five buffer changes to remove the unconjugated SCN-Bz-DTPA and SCN-Bz-DOTA. After conjugation, the protein concentration was determined spectrophotometrically (Amersham Pharmacia Biotech, Uppsala, Sweden) at 280 nm. The substitution ratio was determined by the labelling of the conjugation mixture with ^111^InCl_3 _(Covidien, Petten, The Netherlands) described by Hnatowich et al. [12]. After incubation at room temperature [RT] for 20 min, quality control was performed on silica-gel instant thin layer chromatography [ITLC] strips (ITLC-SG, Biodex Medical Systems, Inc., Shirley, NY, USA) with sodium citrate, with a pH of 5.5, as the mobile phase (retention factor [*R*_f_] ^111^In-labelled anti-CAIX mAb = 0, *R*_f _^111^In-DTPA or ^111^In-DOTA = 1). The substitution ratio is represented by the percentage of activity with an *R*_f _of 0 when the conjugation mixture is labelled.

### Buffers

Sodium acetate (Merck, Darmstadt, Germany) was dissolved in distilled water (Versol, Lyon, France) to a final concentration of 0.1 M, and the pH was adjusted to 5.5 by titration with 1 M HCl (Merck, Darmstadt, Germany). Ammonium acetate buffer was prepared by mixing equal volumes of 0.2 M acetic acid (Merck, Darmstadt, Germany) and 0.2 M ammonia (Merck, Darmstadt, Germany), and the pH was adjusted to 5.5 by adding 0.2 M acetic acid or 0.2 M ammonia. MES and HEPES (Sigma-Aldrich Corporation, St. Louis, MO, USA) were dissolved in distilled water to a final concentration of 0.1 M, and the pH was adjusted to 5.5 with 1 M NaOH (Merck, Darmstadt, Germany).

### ^111^In-labelling of peptides and antibodies

The labelling of the six compounds with ^111^In was performed 9 days after ^111^In production (The calibration date of ^111^InCl_3 _is 10 days after the production of ^111^InCl_3_, and the expiry date of ^111^InCl_3 _is 11 days after the production of ^111^InCl_3_). The peptides and antibodies were dissolved in metal-free water to a final concentration of 0.1 μg/μl, and 5 μl was added to a 0.1 M NaAc, NH4Ac, MES or HEPES buffer. Five volumes of buffer and one volume of ^111^InCl_3 _(Covidien, Petten, The Netherlands) were added. The reaction mixtures were incubated for 20 min at RT for DTPA-conjugated compounds, at 95°C for DOTA-exendin and DOTA-octreotide or at 45°C for the DOTA-conjugated anti-CAIX antibody. After incubation, Tween80 (Sigma-Aldrich Corporation, St. Louis, MO, USA) was added to a final concentration of 0.1%, and ethylenediaminetetraacetic acid [EDTA] (Sigma-Aldrich Corporation, St. Louis, MO, USA) in 0.25 M NH_4_Ac, with a pH of 5.5, was added to a final concentration of 5 mM to complex unincorporated ^111^In. Quality control was performed on silica-gel ITLC strips with 0.1 M EDTA in 0.1 M NH_4_Ac as a mobile phase (*R*_f _^111^In-labelled compounds = 0, *R*_f _^111^In-EDTA = 1). The maximum SA was determined by correcting the initial SA for the radiochemical purity.

### Effect of ageing of the ^111^InCl_3 _stock on the labelling efficiency of DTPA-exendin-3

DTPA-exendin-3 (0.5 μg) was labelled in triplicate (except for *t *= 14, which is in duplicate) with ^111^In (75 MBq) in 0.1 M NaAc, NH_4_Ac, MES and HEPES, with a pH of 5.5, as described above, from 4 days after the production date (delivery of ^111^InCl_3_) until 14 days after the production date of ^111^InCl_3_. Quality control was performed as described above.

### Effect of the presence of cadmium on the labelling efficiency of DTPA-exendin-3

The effect of cadmium, the decay product of ^111^In, on the radiolabelling was examined by adding increasing amounts of Cd^2+ ^to the labelling mixture of DTPA-exendin-3. CdCl_2 _(Sigma-Aldrich Corporation, St. Louis, MO, USA) was dissolved in 0.1 M Ultrapure HCl (J.T. Baker, Deventer, The Netherlands), and serial dilutions ranging from 10^-1 ^to 10^-7 ^M CdCl_2 _in 0.02 M HCl were prepared. DTPA-exendin-3 (0.5 μg) was labelled with 1.85 MBq ^111^InCl_3 _(at day 9 after ^111^InCl_3 _production) in 0.1 M NaAc, NH_4_Ac, MES and HEPES, with a pH of 5.5, as described above, and various amounts of CdCl_2 _were added simultaneously with ^111^InCl_3 _to amounts ranging from 1 × 10^-3 ^to 9 × 10^4 ^nmol (resulting in final concentrations of Cd^2+ ^ranging from 1 pM to 8.3 μM). The amount of buffer was adjusted for the amount of CdCl_2 _in 0.02 M HCl added (final pH 5.5). The experiment was performed in triplicate for all CdCl_2 _concentrations and all buffers. Quality control was performed as described above.

## Results

### Substitution ratio of DTPA- and DOTA-anti-CAIX

The substitution ratio of DTPA- and DOTA-anti-CAIX was 3 DTPA and 7 DOTA molecules per antibody molecule, respectively.

### Effect of the buffer on the labelling efficiency of DTPA conjugates

**Figure 1:**
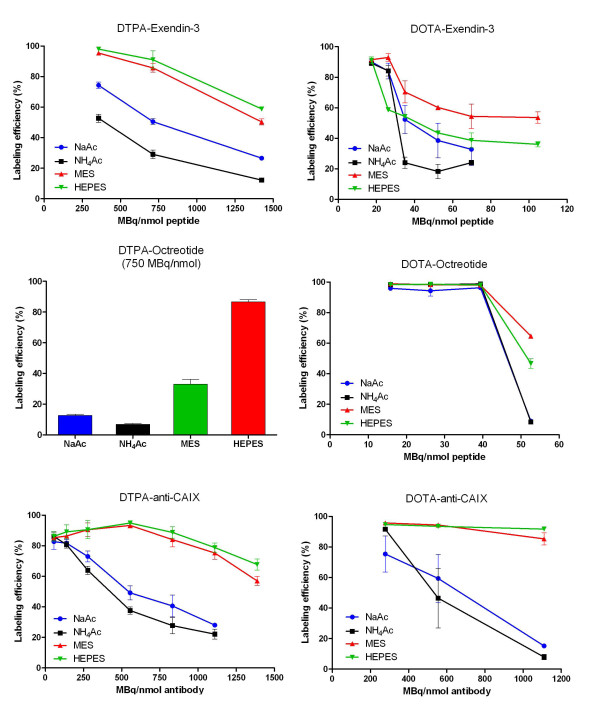
**^111^In-labelling**. ^111^In-labelling of DTPA-exendin-3, DOTA-exendin-3, DTPA-octreotide, DOTA-octreotide, DTPA-anti-CAIX and DOTA-anti-CAIX in 0.1 M NaAc, NH_4_Ac, MES and HEPES buffers.

**Figure 2:**
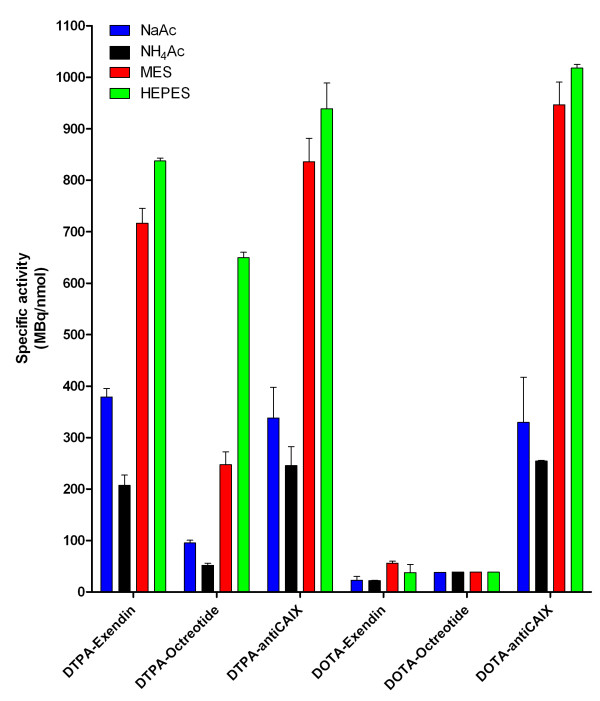
**Maximal specific activities**. Maximal specific activities (in megabecquerel per nanomole) for DTPA-exendin-3, DOTA-exendin-3, DTPA-octreotide, DOTA-octreotide, DTPA-anti-CAIX and DOTA-anti-CAIX in 0.1 M NaAc, NH_4_Ac, MES and HEPES buffers.

The labelling efficiency at different specific activities of DTPA-exendin-3, DTPA-octreotide and DTPA-anti-CAIX in 0.1 M NaAc, NH_4_Ac, MES and HEPES is summarized in Figure 1. The maximum specific activities of the compounds in different buffers were calculated and are shown in Figure 2 and Table 1. Labelling of DTPA-exendin-3 in NaAc buffer resulted in a maximal SA of 379 ± 16 MBq/nmol. The SA was somewhat lower when DTPA-exendin-3 was labelled in NH_4_Ac, 207 ± 20 MBq/nmol. Two- to fourfold higher specific activities were observed when DTPA-exendin-3 was labelled in MES or HEPES (717 ± 29 and 837.3 ± 6 MBq/nmol, respectively). Similar results were observed for the labelling of DTPA-octreotide and DTPA-anti-CAIX (Figures 1 and 2, Table 1).

**Table 1:** Maximal specific activities of DTPA- and DOTA-conjugated compounds

Compound	NaAc (MBq/nmol)^a^	NH_4_Ac (MBq/nmol)^a^	MES (MBq/nmol)^a^	HEPES (MBq/nmol)^a^	Maximum theoretical SA^b ^(GBq/nmol)
DTPA-exendin-3	379 ± 16	207 ± 20	717 ± 29	837 ± 6	1.7
DTPA-octreotide	95 ± 5	52 ± 4	248 ± 24	650 ± 10	1.7
DTPA-cG250	338 ± 60	246 ± 37	835 ± 46	939 ± 50	5.2
DOTA-exendin-3	23 ± 8	22 ± 1	56 ± 4	38 ± 16	1.7
DOTA-octreotide	38 ± 0	39 ± 0	39 ± 0	39 ± 0	1.7
DOTA-cG250	330 ± 87	254 ± 2	947 ± 44	1018 ± 7	12.4

^a^Maximal specific activities (in megabecquerel per nanomole) and the ^b^maximum theoretical SA (in gigabecquerel per nanomole) for DTPA-exendin-3, DOTA-exendin-3, DTPA-octreotide, DOTA-octreotide, DTPA-anti-CAIX and DOTA-anti-CAIX in 0.1-M NaAc, NH_4_Ac, MES and HEPES buffers. The maximum theoretical SA is calculated, assuming that 1 nmol DTPA or DOTA can complex 1 nmol ^111^In.

**Figure 3:**
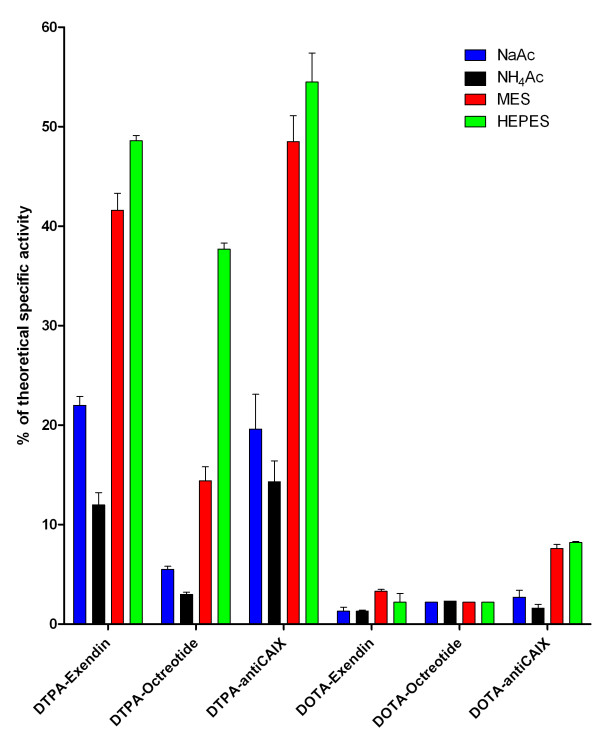
**Percentage of the maximum theoretical SA**. Percentage of the maximum theoretical SA of DTPA-exendin-3, DOTA-exendin-3, DTPA-octreotide, DOTA-octreotide, DTPA-anti-CAIX and DOTA-anti-CAIX in 0.1 M NaAc, NH_4_Ac, MES and HEPES buffers.

When DTPA-exendin-3 was labelled in MES, the SA was 42 ± 2% of the maximum theoretical SA (Figure 3). Labelling of DTPA-exendin-3 in HEPES resulted in a SA that was 49 ± 1% of the maximum theoretical SA and was higher than the SA in acetate buffers (NaAc 22 ± 1% and NH_4_Ac 12 ± 1%). Similar results were obtained for the labelling of the anti-CAIX antibody, whereas the overall complexation of ^111^In by DTPA-octreotide was somewhat lower for all buffers.

### Effect of the buffer on the labelling efficiency of DOTA conjugates

The SA for ^111^In-DOTA-exendin-3 was lower than that of ^111^In-DTPA-exendin-3 (Figure 2 and Table 1). However, the same trend was observed: Labelling in MES and HEPES resulted in higher SA (56 ± 4 and 38 ± 16 MBq/nmol) compared to that in the acetate buffers (NaAc 23 ± 8 MBq/nmol and NH_4_Ac 22 ± 1 MBq/nmol), with the exception that MES performed better than HEPES in these experiments (Figures 1 and 2, Table 1). Also, for DOTA-anti-CAIX, higher SA were observed in MES and HEPES buffer, 947 ± 44 and 1,018 ± 7 MBq/nmol, respectively, versus 330 ± 87 MBq/nmol for NaAc and 254 ± 2 MBq/nmol for NH_4_Ac (Figures 1 and 2, Table 1). No difference in specific activities was observed for DOTA-octreotide (Figures 1 and 2, Table 1).

The complexation of ^111^In by DOTA-conjugated compounds was less efficient than that by DTPA-conjugated compounds (Figure 3). The most efficient complexation of ^111^In was achieved by the labelling of DOTA-anti-CAIX in HEPES buffer, 8.2 ± 0.1% of the DOTA chelates complexed an ^111^In atom. Labelling in HEPES buffer resulted in similar complexation efficiency (7.6 ± 0.4%), whereas labelling in acetate buffers resulted in a three to fivefold reduction in the percentage of DOTA molecules complexed (NaAc 2.7 ± 0.7 and NH_4_Ac 1.6 ± 0.4). Incorporation of ^111^In was also more efficient in HEPES and MES buffer for DOTA-exendin-3, but no differences in complexation efficiency were observed when DOTA-octreotide was labelled.

### Effect of ageing of the ^111^InCl_3 _stock on the labelling efficiency of DTPA-exendin-3

**Figure 4:**
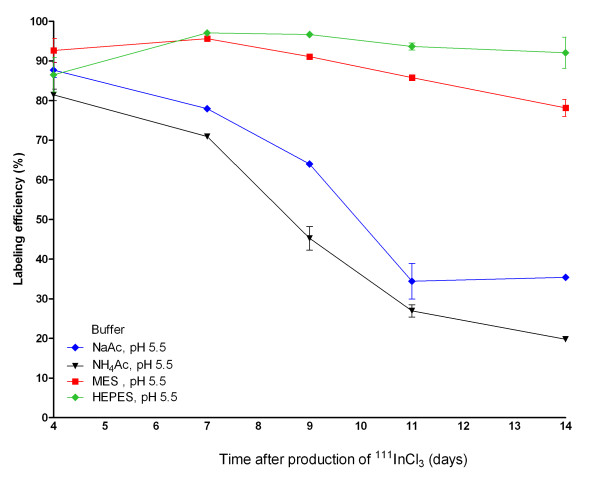
**Radiolabelling of DTPA-exendin-3 with ^111^In**. Radiolabelling of DTPA-exendin-3 with ^111^In at different time points from the production (day 0) of ^111^InCl_3 _for 0.1 M NaAc, NH_4_Ac, MES and HEPES. Asterisk, labelling performed in duplicate; double asterisks, single labelling performed.

**Figure 5:**
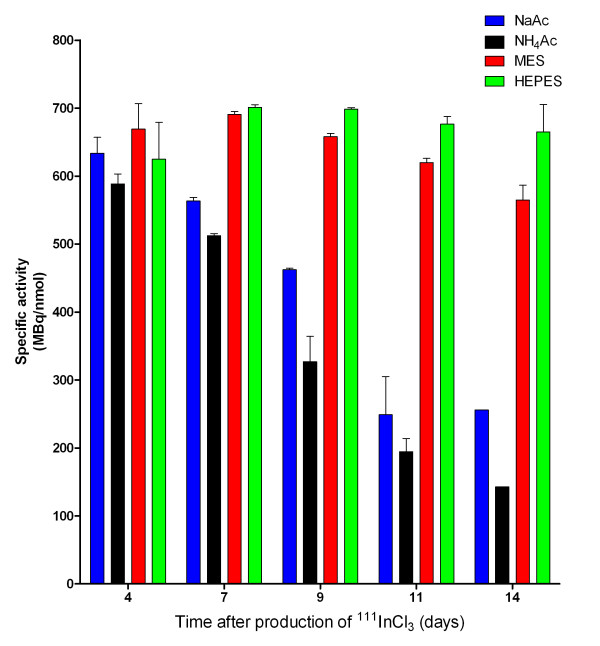
**Maximal SA of ^111^In-DTPA-exendin-3 at several time points after ^111^InCl_3 _production (*t *= 1 day)**. The SA was calculated by the initial SA (712 MBq/nmol) corrected by the labelling efficiency. Asterisk, labelling performed in duplicate; double asterisks, single labelling performed.

The effect of ageing of the ^111^InCl_3 _stock on the labelling efficiency of DTPA-exendin-3 in 0.1 M NaAc, NH_4_Ac, MES and HEPES was investigated, and the results are summarized in Figures 4 and 5. Four days after the production of ^111^InCl_3 _(arrival of ^111^InCl_3 _stock), DTPA-exendin-3 could be labelled with ^111^In with similar labelling efficiency, resulting in similar SA, for all buffers. In NaAc and NH_4_Ac, a reduced labelling efficiency was observed as soon as 7 days after the production of ^111^InCl_3_, decreasing to a labelling efficiency of 34 ± 8% and 27 ± 3% for NaAc and NH_4_Ac, respectively at day 11. Only a minimal decrease in labelling efficiency was observed when the labelling was performed in MES buffer: from 92.6 ± 5.2% at day 4 to 78.3 ± 3.0% at day 14. A decrease in labelling efficiency was not observed up to day 9. The time point of radiolabelling did not have any significant effect on the labelling efficiency or SA when HEPES was used for the radiolabelling. Labelling in HEPES at day 4 resulted in a labelling efficiency of 87 ± 7% with a SA 627 ± 54 MBq/nmol. A labelling efficiency of 92 ± 6% and a SA of 625 ± 54 MBq/nmol were obtained at day 14 after the production date of ^111^InCl_3_. These results were not significantly different from the results obtained 4 days after the production date.

### Effect of the presence of cadmium on the labelling efficiency of DTPA-exendin-3

**Figure 6:**
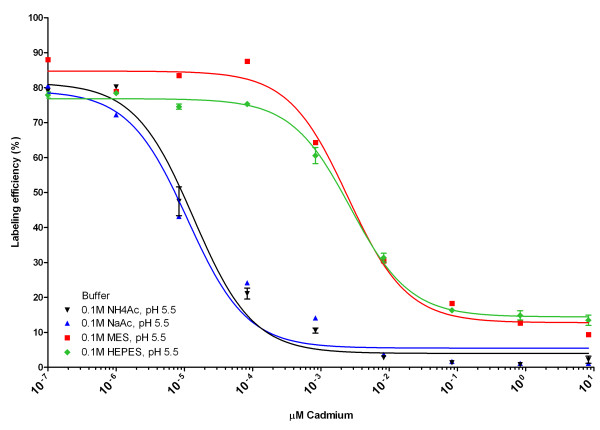
**Effect of cadmium on labelling of ^111^In-DTPA-exendin-3 in 0.1 M NaAc, NH_4_Ac, MES and HEPES**.

In Figure 6 and Table 2 the effect of the Cd^2+ ^concentration in the labelling mixture on the labelling efficiency of DTPA-exendin-3 is summarized. A decrease in labelling efficiency was observed when 1 pM CdCl_2 _was added to the labelling of DTPA-exendin-3 with ^111^In in NaAc and NH_4_Ac, whereas up to 0.1 nM Cd^2+ ^did not affect the labelling efficiency when DTPA-exendin-3 was labelled in MES or HEPES.

**Table 2:** 50% Inhibitory concentration of cadmium on the ^111^In-labelling of DTPA-exendin-3

Buffer	50% inhibitory concentration of Cd^2+ ^(nM)^a^
NaAc	0.011 (0.007 to 0.019)
NH_4_Ac	0.013 (0.010 to 0.019)
MES	2.5 (1.5 to 4.1)
HEPES	2.7 (2.2 to 3.3)

^a^Cadmium concentrations that lead to a 50% reduction in the labelling efficiency of DTPA-exendin-3 in 0.1 M NaAc, NH_4_Ac, MES and HEPES. The 95% confidence interval is indicated in parentheses.

The Cd^2+ ^concentration that lead to a 50% reduction in labelling efficiency was lower in NaAc (0.011 nM, 95% confidence interval 0.007 to 0.019 nM) and NH_4_Ac (0.013 nM, 95% confidence interval 0.010 to 0.019 nM) than that in MES (2.5 nM, 95% confidence interval 1.5 to 4.1 nM) and HEPES (2.7, 95% confidence interval 2.2 to 3.3 nM), indicating that the labelling efficiency was not affected by Cd^2+ ^contamination in MES and HEPES buffer.

## Discussion

High SA of ^111^In-labelled peptides and antibodies is required to administer a tracer dose of peptide or protein, preventing target saturation and/or side effects, while administering high activity doses required for imaging. Acetate buffers are routinely used for the labelling of DTPA- and DOTA-conjugated compounds with ^111^In. Here, we examined the effect of the buffer used during the radiolabelling: HEPES and MES, and compared this with the most commonly used acetate buffers: sodium acetate and ammonium acetate, and showed that an increased SA could be obtained when DTPA- and DOTA-conjugated compounds were labelled in MES or HEPES buffer. Moreover, the labelling efficiency was not affected by Cd^2+ ^concentrations up to 0.1 nM when the labelling was performed in MES and HEPES, whereas a drastic effect was observed when the labelling was performed in acetate buffers. In line with these results, the ageing of the ^111^InCl_3 _stock had only a minor effect on the labelling efficiency 14 days after the production of ^111^InCl_3 _when compounds were labelled in MES and HEPES.

The use of MES as a buffer for radiolabelling resulted in a SA of all DTPA-conjugated compounds that was approximately two to three times higher when compared to radiolabelling in ammonium acetate and sodium acetate, respectively. When HEPES was used, an even higher SA of the DTPA-conjugated compounds was observed: four times higher than the labelling performed in ammonium acetate. The effect was less pronounced when the DOTA-conjugated compounds were labelled with ^111^In. Overall, radiolabelling in HEPES and MES was more efficient than that in acetate buffers in most cases and at least as efficient as in the case of DOTA-octreotide. Labelling of DOTA-conjugated compounds resulted in 5 to 20 times lower SA than that of DTPA-conjugated compounds. Most likely, this is due to the interference of contaminating metals with DOTA chelation, which might play a role to a lesser extent when labelling DTPA-conjugated compounds.

The decay product of ^111^In, ^111^Cd, can also be chelated by DTPA or DOTA, and it is therefore expected that the complexation of ^111^In is less efficient over time due to increasing amounts of Cd^2+^. Indeed, this phenomenon was observed when sodium acetate and ammonium acetate were used for the ^111^In-labelling of DTPA-exendin. Lower labelling efficiencies were observed as early as 7 days after the production of ^111^InCl_3_, and threefold lower SA were obtained when the labelling was performed with ^111^InCl_3 _11 days after the production date. This effect was not observed for the labelling of DTPA-exendin-3 in MES and HEPES with a maximal decrease in SA of 10% and 4% at day 11, respectively. Even the decrease in SA 14 days after the calibration date of ^111^InCl_3 _was not more than 18% for ^111^In-labelling in MES and 5% for HEPES. These latter results could explain the differences in SA of the six compounds used in this study since the labelling of these compounds was performed with ^111^In 9 days after production. Generally, ^111^InCl_3 _is used from 7 to 11 days after the production day, which could lead to reduced specific activities at later time points when acetate buffers are used. To overcome this problem, HEPES or MES buffer could be used for radiolabelling, with high specific activities at time points up to 14 days after ^111^InCl_3 _production. This could have an impact on experiment planning since experiments which require high-SA-labelled compounds are only available early after ^111^In production when acetate buffers are used, whereas the time point is not relevant when MES or HEPES is used. These results suggest that increasing amounts of Cd^2+ ^contamination, due to the ageing of the ^111^InCl_3 _stock, do not influence the labelling of DTPA-conjugated compounds when MES and HEPES are used as a buffer for radiolabelling.

The suggested effect of cadmium on the ^111^In-labelling of DTPA-conjugated compounds was confirmed when increasing amounts of Cd^2+ ^were added to the ^111^In-labelling mixture of DTPA-exendin. In HEPES and MES buffer, a 100-fold higher amount of cadmium could be added to the labelling mixture without reducing the labelling efficiency than in acetate buffer. The decreased labelling efficiency at low concentrations of cadmium might be due to the efficient formation of coordination complexes of Cd^2+ ^with acetate, allowing efficient 'transchelation' of Cd^2+^, whereas no coordination complex with HEPES or MES is formed [9], and transchelation of Cd^2+ ^to DTPA or DOTA is less efficient.

It has been postulated that coordination complex formation of ^111^In with acetate buffers is necessary for efficient labelling of DTPA- and DOTA-conjugated compounds [13] since it is assumed that the coordination complex formation prevents the formation of insoluble ^111^In-hydroxide. This study suggests that coordination complex formation of the buffer with ^111^In is less important for efficient labelling of DTPA- and DOTA-conjugated compounds since the labelling in the weakly coordinating buffers MES and HEPES was more efficient than that in acetate buffers in most cases or at least equivocal in the case of DOTA-octreotide.

Breeman et al. described the effect of contaminants on the labelling of DOTA-octreotide with ^111^In, ^177^Lu and ^90^Y, and found a similar result of the effect of cadmium contamination on the radiolabelling [14]. The labelling procedures described in the latter study were performed in sodium acetate, and these findings are in line with the findings in our study, where a pronounced effect of Cd^2+ ^on the labelling of DTPA-exendin-3 is observed when sodium acetate is used as buffer for radiolabelling.

The purification of ^111^InCl_3 _by an anion exchange method was described to improve the labelling of DTPA- and DOTA-conjugated compounds caused by the removal of contaminants, mainly Cd^2+^, present in the ^111^InCl_3 _solution [15]. By using HEPES or MES buffer for the labelling of the compounds, this could omit a time-consuming purification method.

## Conclusions

We showed improved labelling of DTPA- and DOTA-conjugated peptides, proteins and antibodies with ^111^In when HEPES or MES buffer was used for radiolabelling. The enhanced labelling efficiency could be due to the reduced competitive chelation of cadmium, the decay product of ^111^In. When ^111^In-labelling of DTPA- and DOTA-conjugated compounds is performed in MES or HEPES, ^111^In-labelled compounds can be produced with high specific activities regardless from the time point after ^111^In production.

## Competing interests

The authors declare that they have no competing interests.

## Authors' contributions

MB and LJ performed the ^111^In-labelling studies. MB, LJ, MG and OCB participated in the study design and coordination. MB drafted the manuscript. LJ, MG, WJGO and OCB proofread the manuscript. All authors read and approved the final manuscript.
